# Supplementary material for: Archaeogenomic evidence reveals prehistoric matrilineal dynasty
Source: Nat Commun. 2017 Feb 21;8:14115. doi: 10.1038/ncomms14115 (PMC5321759; doi:10.1038/ncomms14115)
Supplement: Supplementary Information — Supplementary Figures, Supplementary Tables, and Supplementary Notes [file ncomms14115-s1.pdf]

Supplementary Figure 1 - Stratigraphic model and calibrated AMS ^{14}C results for Pueblo Bonito Room 33 crania.

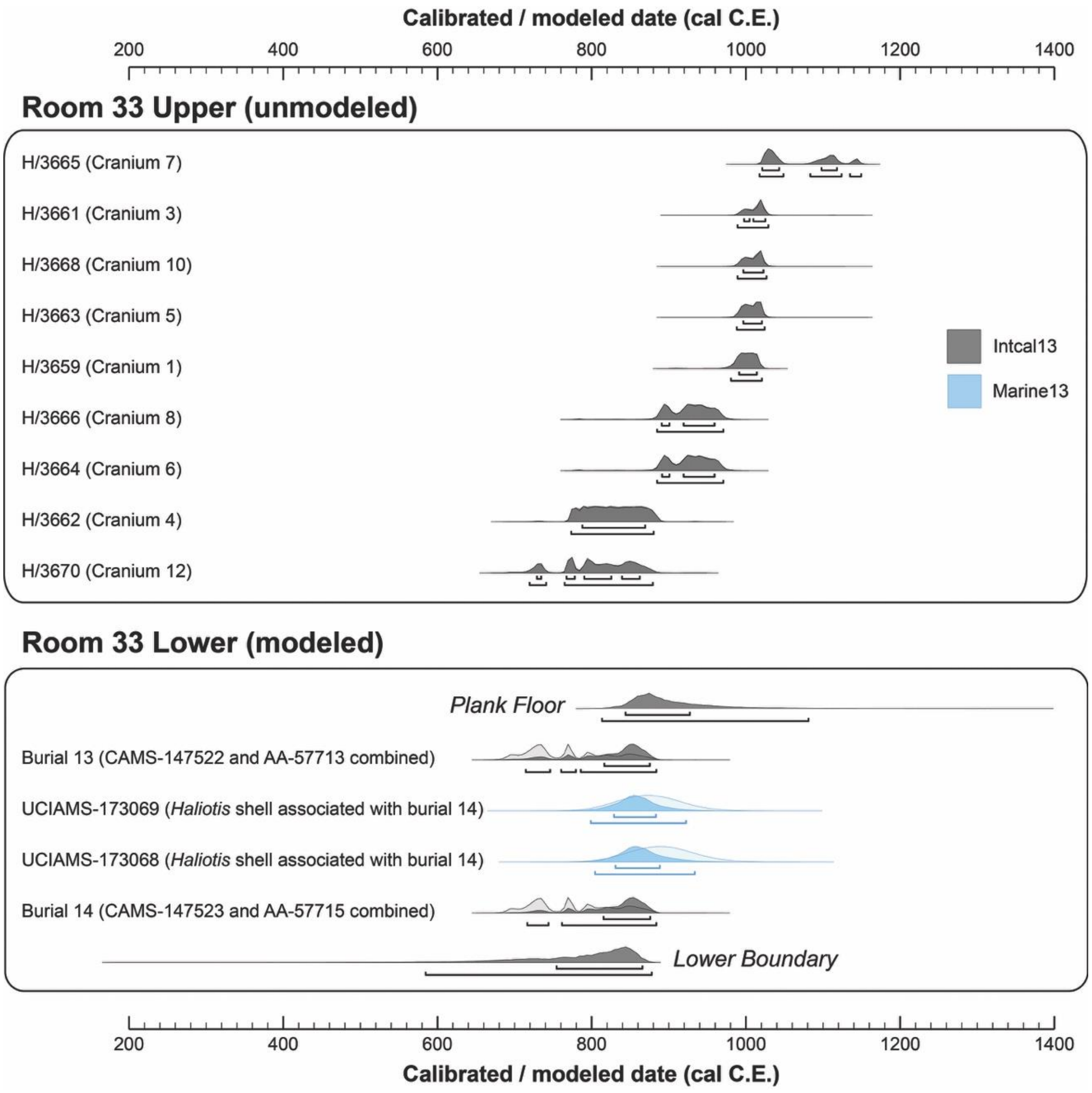

Supplementary Figure 2 - Calibrated AMS ^{14}C dates on 11 human crania from Pueblo Bonito Room 33 plotted on the IntCal13 curve, show the effects of plateau and reversals on the resulting distributions and overall temporal patterning of the calibrated data.

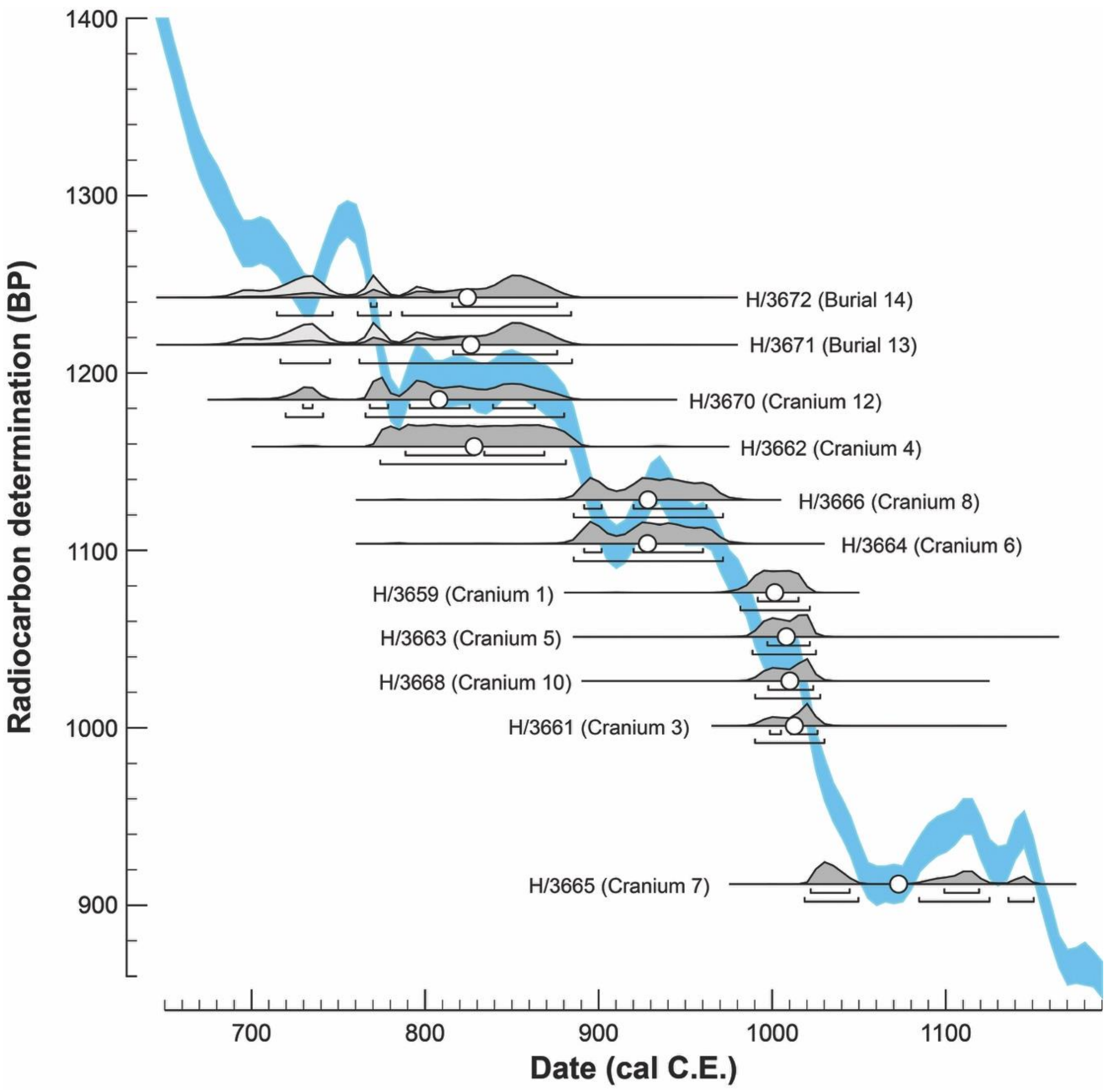

Supplementary Figure 3 - Frequency of 210 simulated conventional ^{14}C ages (grey) from 750 to 1250 CE showing periods likely to be over- and under-represented in a random dating sample, versus the observed ^{14}C ages of 11 human crania from Pueblo Bonito Room 33 (yellow).

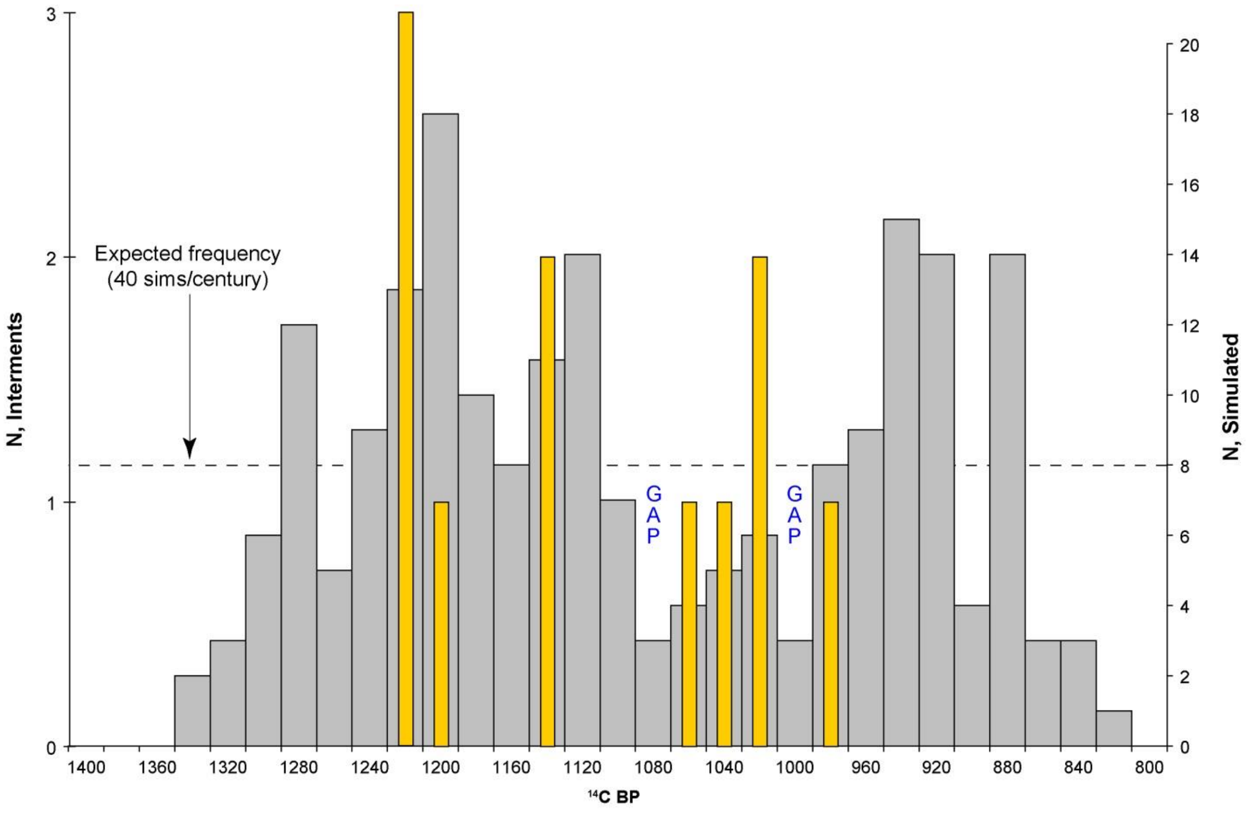

Supplementary Figure 4 - Ancient DNA misincorporation profiles for Pueblo Bonito Room 33 samples.

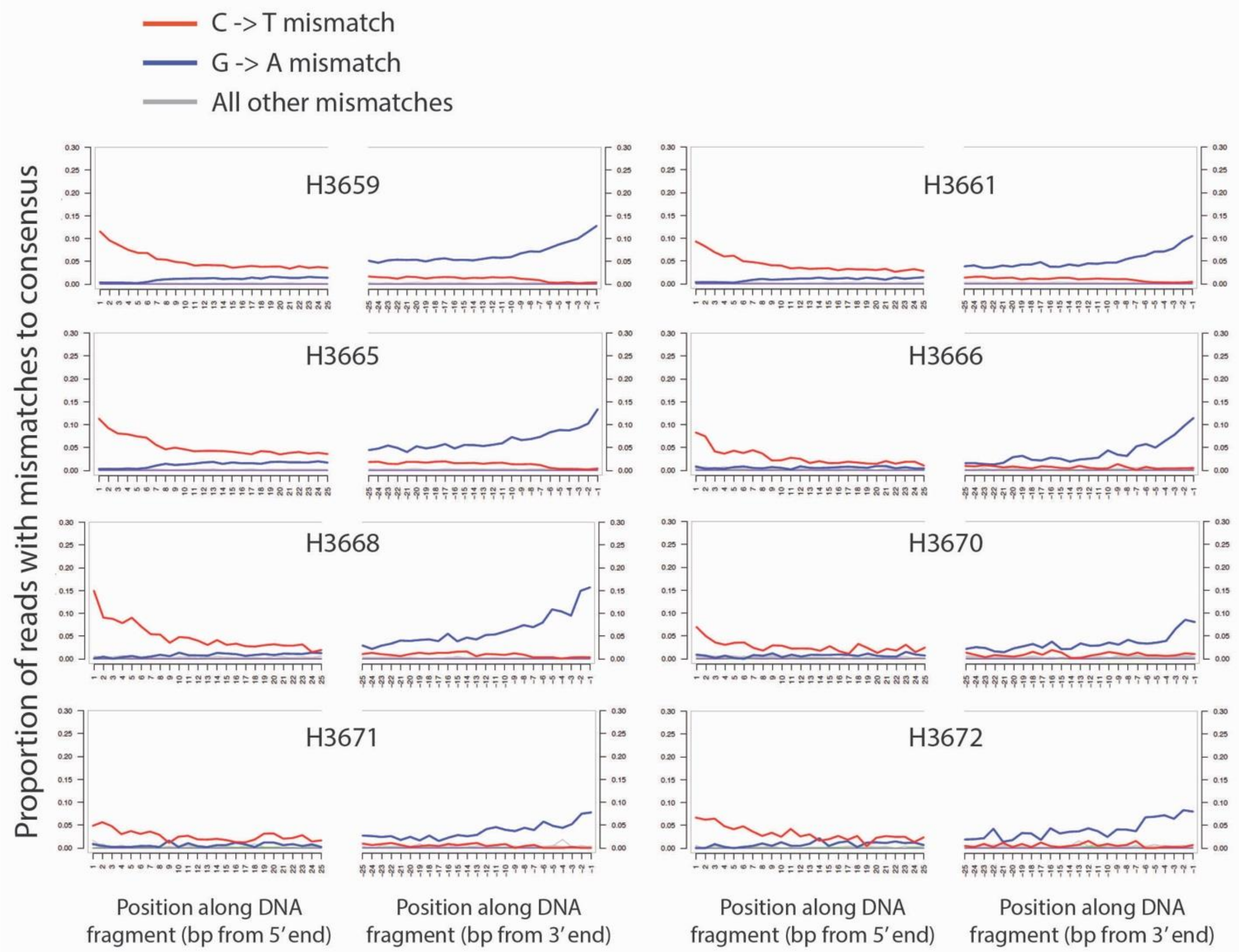

Supplementary Figure 5 - Pairwise distance matrix with Chaco region ancient DNA sequences from this study and 171 complete modern Native American mtDNA genomes.

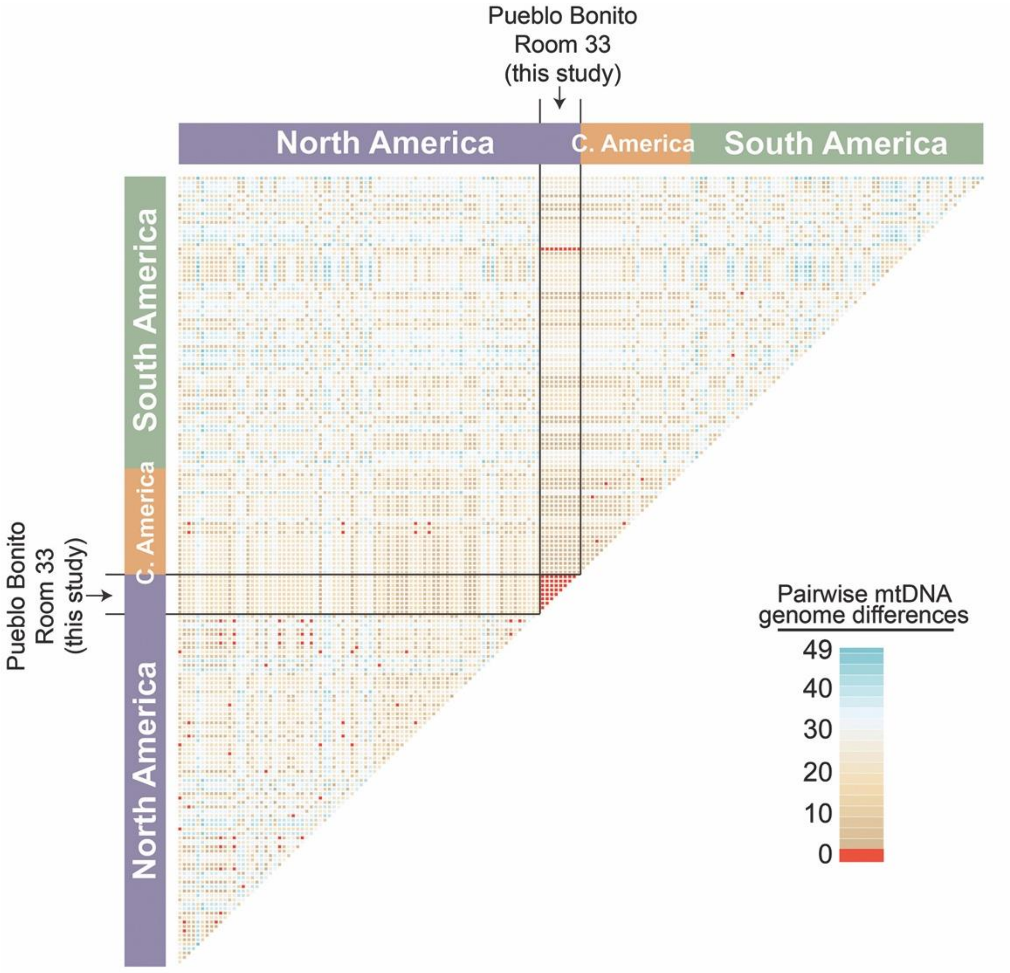

Supplementary Figure 6 - Estimates and 95% confidence intervals for the relatedness coefficient r in pairs of Chaco individuals. The “Loci” column provides the number of SNPs covered by at least one sequence read in both of the two individuals being compared in that pairwise analysis.

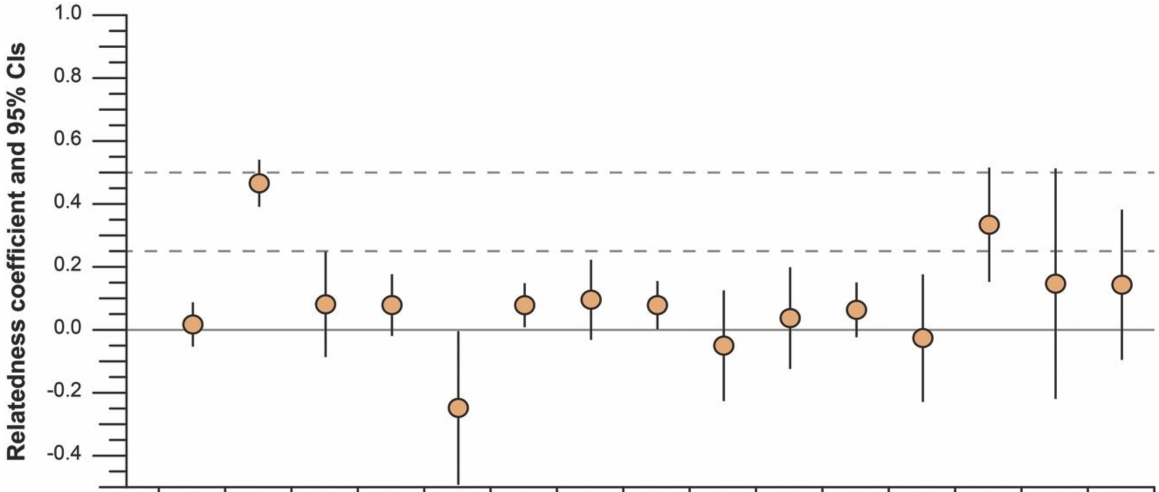

Pair	1 and 3	1 and 7	1 and 8	1 and 10	1 and 12	3 and 7	3 and 8	3 and 10	3 and 12	7 and 8	7 and 10	7 and 12	8 and 10	8 and 12	10 and 12
Loci	11,724	7,983	2,035	5,914	1,059	13,446	3,301	9,849	1,765	2,305	7,397	1,269	1,698	370	949

Supplementary Figure 7 – Illustration of genetic distance between generations of a typical matrilineage (members are in red): In genetic analysis, lineal ties separated by one generation (mother [e] with her daughter or son [1]) are first-degree relations; sibling relationships are also first-degree. Individuals separated by two generations (a grandmother with grandson or granddaughter or individuals who are first generation descendants of a lineage male and a non-lineage female such as an aunt and a niece or nephew [2]) are second-degree relations, and those separated by three generations are third-degree relations (Great grandmother-great grandson or granddaughter [3].

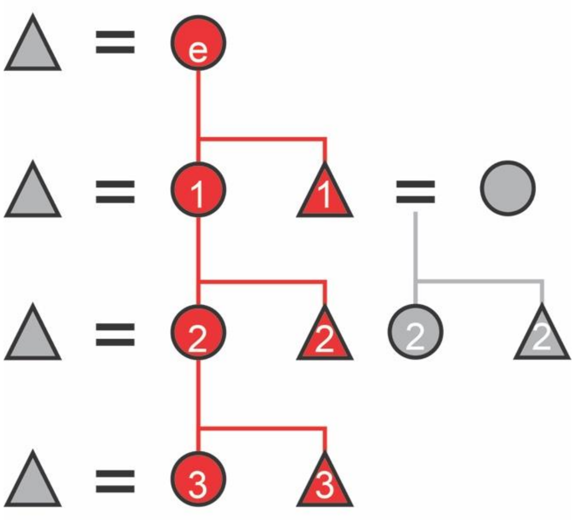

Supplementary Table 1 - Stable isotope, %C, %N, and conventional radiocarbon results for the directly dated crania from Pueblo Bonito Room 33.

Description	PSU#	UCIAMS#	Lab ID	$\delta^{13}C$ (‰ VPDB)	$\delta^{15}N$ (‰ Atm N_2)	%C	%N	C:N	^{14}C Age BP
Cranium 1	6214	145217	SP3659	-8.6	12.2	42.7	16.0	3.11	1045±15
Cranium 3	6223	145226	SP3661	-8.8	11.5	42.7	16.0	3.11	1015±15
Cranium 4	6215	145218	SP3662	-7.1	13.3	43.9	16.4	3.13	1195±15
Cranium 5	6216	145219	SP3663	-9.5	11.9	43.1	16.1	3.13	1025±15
Cranium 6	6217	145220	SP3664	-8.0	12.3	42.9	16.0	3.13	1130±15
Cranium 7	6218	145221	SP3665	-8.6	11.4	46.8	17.6	3.11	970±15
Cranium 8	6280	148198	SP3666	-8.4	12.7	42.0	15.6	3.14	1130±15
Cranium 10	6224	145227	SP3668	-8.9	11.1	43.0	16.1	3.11	1020±15
Cranium 12	6219	145222	SP3670	-7.2	12.8	40.8	15.3	3.12	1220±15

Supplementary Table 2 - Calibrated ages for unmodeled (prior) and modeled (posterior) dates and boundary estimates.

Description	14C Age BP	Unmodeled cal C.E.	%	Modeled cal C.E.	%	Wtd mean	±
Cranium 7	970±15	1020–1150	95.4			1070	40
Cranium 3	1015±15	990–1030	95.4			1010	10
Cranium 10	1020±15	990–1030	95.4			1010	10
Cranium 5	1025±15	990–1030	95.4			1010	10
Cranium 1	1045±15	980–1020	95.4			1000	10
Cranium 6	1130±15	890–970	95.4			930	30
Cranium 8	1130±15	890–970	95.4			930	30
Cranium 4	1195±15	770–880	95.4			830	30
Cranium 12	1220±15	720–880	95.4			810	40
Plank floor (boundary)				810–1080	95.4	910	90
Burial 13 (combined)	1245±25<br>1209±40	690–880	95.4	720–890	95.4	830	40
Haliotis shell	1730±20	810–980	95.4	810–940	95.4	870	30
Haliotis shell	1745±20	800–960	95.4	800–920	95.4	860	30
Burial 14 (combined)	1240±25<br>1213±40	689–880	95.4	720–890	95.4	830	40
Lower boundary				590–880	95.4	770	100

Supplementary Table 3 - Relatedness analysis.

Pair (cranium or burial number)	Loci	*r*	*r* (lower 95% CI)	*r* (upper 95% CI)	Relatedness
1 and 3	11,724	0.0171	-0.0528	0.0870	
1 and 7	7,983	0.4658	0.3913	0.5402	First-degree
1 and 8	2,035	0.0812	-0.0862	0.2486	
1 and 10	5,914	0.0788	-0.0189	0.1765	
1 and 12	1,059	-0.248	-0.4923	-0.0043	
3 and 7	13,446	0.0784	0.0087	0.1481	
3 and 8	3,301	0.0958	-0.0308	0.2224	
3 and 10	9,849	0.0786	0.0021	0.1551	
3 and 12	1,765	-0.050	-0.2259	0.1250	
7 and 8	2,305	0.0374	-0.1238	0.1986	
7 and 10	7,395	0.0636	-0.0231	0.1502	
7 and 12	1,269	-0.026	-0.2286	0.1759	
8 and 10	1,698	0.3342	0.1527	0.5156	First- or second-degree
8 and 12	370	0.1467	-0.2193	0.5127	
10 and 12	949	0.1434	-0.0951	0.3819	

Supplementary Note 1 - Matrilineal kinship typically correlates with matrilocal residence. At marriage, a husband relocates from his natal household and joins his wife's family, resulting in the multi-generation presence of the female members of the matrilineage in the same residential complex. Matrilineages often control or are associated with the key components of production and power, including land, ritual paraphernalia, the knowledge needed to properly conduct important ceremonies, and thus rank or status. They also often both prescribe and proscribe marriage ties among different social groups, often defining the set of individuals who a person cannot marry and sometimes specifying those who can or should be married.

Supplementary Note 2 - OxCal Code for Room 33 Bayesian Chronological Model.

```
Plot()
 {
  Sequence()
  {
   Boundary("Lower Boundary");
   Phase("Room 33 Lower")
   {
    R_Combine("Burial 14")
    {
     R_Date("CAMS-147523 Cranium 14", 1240,25);
     R_Date("AA-57715 Cranium 14", 1213,40);
    };
    Curve("Oceanic","Marine13.14c")
    {
     Reservoir(234,23);
    };
    R_Date("UCIAMS-173068", 1730, 20);
```

```
    R_Date("UCIAMS-173069", 1745, 20);
    Curve("Atmospheric","IntCal13.14c")
    {
    };
    R_Combine("Burial 13")
    {
     R_Date("CAMS-147522 Cranium 13", 1245,25);
     R_Date("AA-57713 Cranium 13", 1209,40);
    };
   };
   Boundary("Plank Floor");
  };
  //Room 33 Upper (unmodeled)
  R_Date("UCIAMS-145222 Cranium 12", 1220,15);
  R_Date("UCIAMS-145218 Cranium 4", 1195,15);
  R_Date("UCIAMS-145220 Cranium 6", 1130,15);
  R_Date("3666 Cranium 8", 1130,15);
  R_Date("UCIAMS-145217 Cranium 1", 1045,15);
  R_Date("UCIAMS-145219 Cranium 5", 1025,15);
  R_Date("UCIAMS-145227 Cranium 10", 1020,15);
  R_Date("UCIAMS-145226 Cranium 3", 1015,15);
  R_Date("UCIAMS-145221 Cranium 7",970,15);
 };
```
